# Assessing the Moderating Effect of the End User in Consumer Behavior: The Acceptance of Technological Implants to Increase Innate Human Capacities

**DOI:** 10.3389/fpsyg.2016.00132

**Published:** 2016-02-22

**Authors:** Jorge Pelegrín-Borondo, Eva Reinares-Lara, Cristina Olarte-Pascual, Marta Garcia-Sierra

**Affiliations:** ^1^Departamento de Economía y Empresa, Universidad de La RiojaLogroño, Spain; ^2^Departamento de Economía y Empresa, Universidad Rey Juan CarlosMadrid, Spain; ^3^Institute of Environmental Science and Technology, Universitat Autònoma de BarcelonaBarcelona, Spain

**Keywords:** consumer behavior, technology acceptance, technological implants, insideables, cognitive factors, affective factors, subjective norm

## Abstract

Today, technological implants are being developed to increase innate human capacities, such as memory or calculation speed, and to endow us with new ones, such as the remote control of machines. This study's aim was two-fold: first, to introduce a Cognitive-Affective-Normative (CAN) model of technology acceptance to explain the intention to use this technology in the field of consumer behavior; and second, to analyze the differences in the intention to use it based on whether the intended implant recipient is oneself or one's child (i.e., the moderating effect of the end user). A multi-group analysis was performed to compare the results between the two groups: implant “for me” (Group 1) and implant “for my child” (Group 2). The model largely explains the intention to use the insideable technology for the specified groups [variance explained (*R*^2^) of over 0.70 in both cases]. The most important variables were found to be “positive emotions” and (positive) “subjective norm.” This underscores the need to broaden the range of factors considered to be decisive in technology acceptance to include variables related to consumers' emotions. Moreover, statistically significant differences were found between the “for me” and “for my child” models for “perceived ease of use (PEU)” and “subjective norm.” These findings confirm the moderating effect of the end user on new insideable technology acceptance.

## Introduction

Companies and research institutions are currently developing technological implants (insideables) both to increase innate human capacities (Technological Implants to Increase Innate Capacities, T3ICs), such as memory (Berger et al., 2011; Cohen, 2013), and to endow us with new ones, such as the remote control of machines (Regalado, 2015). The fact that this technology could a priori be implanted in healthy people for the sole purpose of enhancing their senses is controversial, and many people even have ambivalent opinions (Olarte-Pascual et al., 2015). For some, the integration of technological implants is considered a “quantum leap” for the species that will allow reasonable people to enhance their capacities to the extent that technology allows (Selinger and Engström, 2008). For others, this technology triggers fear of dehumanization (Lai, 2012). Although not yet quite a reality, T3ICs will most likely be available for future generations. The biomedical engineer Ted Berger, developer of the prosthesis for restoring memory, has noted that such implants will be available for our children in the near future (Berger et al., 2011). With regard to their use in children, Apple co-founder Steve Wozniak has noted that the intention to use T3ICs on one's children may be greater than that to use them on oneself (Jáuregui, 2014) and that, while he “would like to remain natural himself,” he would want his children to have them “if in a few years other kids (…) will have certain advantages thanks to technology.” Nevertheless, public acceptance of this new insideable technology, the subject of the current paper, has not yet been investigated in academic research, whereas the ethical and moral implications of T3ICs have (Schermer, 2009).

With this in mind, the aim of this study was two-fold: first, to introduce a Cognitive-Affective-Normative (CAN) model of technology acceptance to explain the intention to use of this insideable technology in the field of consumer behavior; and second, to analyze the differences in the intention to use it based on whether the intended implant recipient is oneself or one's child (i.e., the moderating effect of the end user). The CAN model was tested on two groups: “T3ICs for me” (Group 1) and “T3ICs for my child” (Group 2).

In conducting research on the acceptance of new technologies, many researchers build on variables from previous models that have proved influential to technology acceptance (e.g., Hameed et al., 2012). In this vein, the Technological Acceptance Model (TAM) variables “perceived usefulness (PU),” “perceived ease of use (PEU),” and “(positive) social norms” positively affects the intention to use a new technology (Davis, 1989; Davis et al., 1989; Venkatesh and Davis, 2000). These variables consistently explain a substantial part (~40%) of the variance in the intention to use innovative technologies, as demonstrated in several studies (Venkatesh and Davis, 2000). The CAN model is based on these previous models of technology acceptance, namely the TAM (Davis, 1989; Davis et al., 1989), the TAM2 (Venkatesh and Davis, 2000), and their extensions via the Unified Theory of Acceptance and Use of Technology (UTAUT and UTAUT2), which include the effect of social influence (Venkatesh et al., 2003, 2012). The CAN model includes the cognitive variables “PU” and “PEU,” as well as the normative variable “subjective (or social) norm.” The literature has recognized the influence of normative factors on people's attitude, intention, and behavior (Fishbein and Ajzen, 1975; Bagozzi, 2000; Venkatesh et al., 2012). The latter may also play a key role in assessing implantation, especially in children, by capturing parents' moral concerns. Indeed, in the field of pediatric surgery, meta-analytic results show that cognitive (i.e., personal factors, preferences), affective and normative factors, namely the opinions of other community members, do influence parent's consent to implantation (Lipstein et al., 2012).

However, the CAN model introduces a novel extension with respect to the TAM and UTAUT models: the inclusion of the affective variables “positive emotions,” “negative emotions,” and “anxiety.” The benefits of including both cognitive and affective factors in order to better understand subjects' assessments of products has been widely acknowledged in the literature (Holbrook and Hirschman, 1982; Shiv and Fedorikhin, 1999; Campbell, 2007; Bigné et al., 2008; Levav and McGraw, 2009; Zielke, 2011).

There are no previous references specific to the acceptance of T3ICs. The closest background literature are studies contrasting the acceptance of implantable medical technology and physical implants for medical or cosmetic reasons. These contexts offer some evidence that can be interpreted as an indication of the possible acceptance of T3ICs. Technological implants to compensate for physical impairments, e.g., peacemakers or cochlear implants to assist children with hearing disabilities, are widely accepted and their use is widespread (Hill and Sawaya, 2004; Rosahl, 2004; Schermer, 2009; Pray and Jordan, 2010). Likewise, the use of physical implants for reasons other than improving one's health status, such as the incorporation of physical implants for breast augmentation (i.e., augmentation mammoplasty), seems to be accepted as well, at least in adults. Many people have already chosen to modify their body to match socially-accepted beauty standards (Adams, 2010) and increase their seductive capacities (Lawton, 2004). As for physical implants for cosmetic reasons, in the US, 4% of all cosmetic surgeries performed in 2014 were performed on patients between the ages of 13 and 19 (American Society of Plastic Surgeons, 2014). In Spain, 10% of all cosmetic surgeries where performed on patients under the age of 18 (Sahuquillo, 2008). Some interventions are due to true pathologies, but not all. Indeed, the Spanish Society of Plastic Reconstructive and Aesthetic Surgery (SECPRE in Spanish) advises against unnecessary cosmetic interventions in minors (Sahuquillo, 2008). It is noteworthy that in most countries minors need the consent of their parents to undergo surgery, although in specific cases, minors over the age of 16 are allowed to decide for themselves. For some people implantation is only considered desirable if it addresses medical issues (impairments due to accidents or illness), not when it is performed for the purpose of beautification (Schaar and Ziefle, 2011).

## Model variables, hypotheses, and multi-group comparison

The CAN model accounts for the influence of cognitive factors (“PU,” and “ease of use”), affective factors (“positive emotions,” “negative emotions,” and “anxiety”) and normative factors [“subjective (or social) norm”] on consumer behavior. The following subsections describe the model variables and underlying hypotheses, including the hypothesis that the decision to undergo implantation is moderated by whether the T3IC is “for me” or “for my child.”

### Models of technology acceptance: cognitive and normative variables

The cognitive variables “PU” and “PEU” are more or less self-explanatory. The former refers to how the technology is perceived to help the user enhance his or her performance, whereas the latter refers to how its use is perceived to be “free of effort” (Davis, 1989). Their influence on the intention to use implants in adults has been proven with regard to physical implants for cosmetic surgery (Adams, 2010), as well as technological implants to address health issues, such as submammary defibrillators, cardiac resynchronization therapy devices, cardioverter defibrillators, or pacemakers (Giudici et al., 2010). “PU” also plays a key role in the intention to use cochlear implants in children (Christiansen and Leigh, 2004; Christie and Bloustien, 2010). These studies support the idea that most parents have high expectations that cochlear implants will help their children improve their verbal communication, educational options, and emotional well-being, often beyond the standard level among the deaf population (Li et al., 2003).

In addition to utilitarian factors, other factors also influence the decision to undergo implantation. Most et al. (2007) highlighted the importance of family environment in attitudes toward cochlear implants. In the same vein, Hyde et al. (2010) noted that parents often found the information provided by professionals insufficient to judge the implications of cochlear implants. In making such a decision, most parents consult other families with implanted children, and children with implants themselves, and highly value their support and the information they provide about their own personal experiences (see also Fitzpatrick et al., 2008).

With regard to body modification for strictly aesthetic purposes, Adams (2010) and Javo and Sørlie (2010) also established the influence of family and friends on the decision to undergo cosmetic surgery in adults. Additionally, for this area of surgery in particular, the social pressure to maintain a youthful and attractive image seems to be crucial (von Soest et al., 2006; Dorneles de Andrade, 2010). The variable “subjective (or social) norm” captures the influence of others' opinion on one's choices. Social norms are expected to play a key role in assessments of potential implantation, especially implantation in children.

Based on the aforementioned observations regarding TAM models, and findings in the therapeutic field, the following hypotheses were proposed with regard to T3ICs:

H1. The perceived usefulness of T3ICs positively affects the intention to use them.
H2. The perceived ease of use of T3ICs positively affects the intention to use them.
H3. A favorable subjective norm regarding the use of T3ICs positively affects the intention to use them.

### Affective variables

A novel extension of models is proposed here in order to capture the effect of emotions on the acceptance of new technologies. Both medical advances in transplant technology, such as organ transplants from animals, and the integration of technological devices have led to the perception that the body is modifiable (Christie and Bloustien, 2010), which, in turn, can generate apprehension and anxiety (Buchanan-Oliver and Cruz, 2011) and fear of dehumanization (Lai, 2012). With regard to young people's perceptions, Schaar and Ziefle (2011) analyzed, through qualitative methods, benefits, and fears regarding four implantable medical devices known to different degrees and entailing different levels of surgical risk for the patient: pacemakers, cochlear implants, medical chips, and deep brain stimulation (or “brain pacemakers”) for the treatment of Parkinson tremor and paralysis. The results showed that, when deciding, young people also make a trade-off between the perceived benefits and risk or fears. Moreover, there was a negative relationship between self-reported technical literacy and risk perception triggered by unspecified concerns and fears resulting from the lack of knowledge of these implants and of technology in general.

With regard to implanting one's children, numerous studies have shown that the process of deciding on cochlear implantation is stressful for the parents of deaf children (Richter et al., 2000; Spahn et al., 2001; Weisel et al., 2006; Zaidman-Zait, 2008). Moreover, parental stress during the pre-examination stage seems to be relatively higher for those with children who are still verbally competent (i.e., borderline cases for cochlear implantation), and for whom this option is therefore not self-evident (Burger et al., 2005). Nevertheless, marked relief is experienced after the initial fitting of the cochlear implant. That is, over time, parents adapt to the new situation and begin to perceive the benefits and adjust their expectations accordingly.

All in all, this evidence supports the inclusion of affective variables in the model of technology acceptance. Doing so enables it to distinguish between emotions that stimulate action, namely implantation, and emotions that inhibit or change the course of action (Oliver et al., 1997; O'Neill and Lambert, 2001; White and Yu, 2005; Cohen et al., 2006). In general, actions associated with positive emotions are evaluated favorably, whereas actions triggering negative emotions are evaluated unfavorably (Bagozzi et al., 1999; Mano, 2004). There is also a natural tendency to avoid decisions that generate bad feelings (Elliott, 1998; Schwarz, 2000; Han et al., 2007).

Using the multidimensional structure of affect (Watson et al., 1988) as a reference, the following hypotheses were proposed:

H4. Positive emotions toward T3ICs positively affect the intention to use them.
H5. Negative emotions toward T3ICs negatively affect the intention to use them.
H6. Feelings of anxiety toward T3ICs negatively affect the intention to use them.

### The user moderating effect: T3ICs “for me” vs. T3ICs “for my child”

According to Chorney et al. (2015), there is a considerable level of decisional conflict when making a decision about surgical treatment for one's child. Hyde et al. (2010) showed how parents found it very difficult to come to a decision on cochlear implantation for children with substantial residual hearing, due to uncertainty regarding improvement. These parents are under greater pressure to make decisions and take on responsibility, and are thus more severely affected by nervous stress (affective factors; Burger et al., 2005). Nevertheless, Li et al. (2004) found that two thirds of the parents of children who were eligible for cochlear implantation were actually considering it. Parents whose children were eligible but who did not consider implantation prioritized bilingual success (verbal and sign language). Indeed, parents might encounter great social pressure in favor of cochlear implantation as opposed to the alternative: letting their child live a “deaf life” (Li et al., 2003; Hyde and Power, 2005; Fitzpatrick et al., 2006). A parallel can be drawn between consenting for children to increase their innate capacities or letting them develop naturally. All in all, the evidence suggests that the factors influencing the decision to undergo implantation and the intensity of their effect may differ depending on whether the intended implant recipient is oneself or one's child.

Notably, affect and normative factors can be relatively more important when deciding for one's children. In a meta-analysis, Lipstein et al. (2012) concluded that a variety of factors influence parents' decisions in the field of pediatric surgery, including personal factors, emotions, and the opinions of other community members. In the same vein, Li et al. (2004) found that aside from medical recommendations, parents' values, beliefs, and expectations about the outcomes of implantation influence the decision to allow their children to undergo cochlear implantation. Moreover, these factors are particularly relevant when parents are considering an early intervention, that is, when there is not enough information confirming that implants are the option that will yield the best outcomes. Indeed, despite the considerable achievements of cochlear implants, this technology still raises questions and poses conflicts and difficulties among parents of children with hearing impairments. The refusal of some parents to have their children implanted may be due to the possibility that children's hearing expectations will remain unfulfilled, which, in turn, could affect their self-esteem (Most et al., 2007). That is, they are seeking to protect their children from disappointment. In addition, Most et al. also demonstrated the existence of a social group identity among some adult deaf people. These people fear that cochlear implantation will lead to a loss of this identity in young deaf people, with no guarantee that the implant will even work as expected. At the opposite extreme, cosmetic surgery in children is sometimes permitted by parents. In many cases, the aim is to achieve not a normal appearance, but an outstanding one (i.e., to look better than the average) (Gilbert, 2009). Accordingly, the following hypothesis was proposed, under the assumption that it can affect all key relationships specified in the model:

H7. The intention to use T3ICs is moderated by whether the T3ICs are “for me” or “for my child,” which involves differences in the explanatory variables affecting the intention to use T3ICs and the intensity of their effect.

### The conceptual model

**Figure 1:**
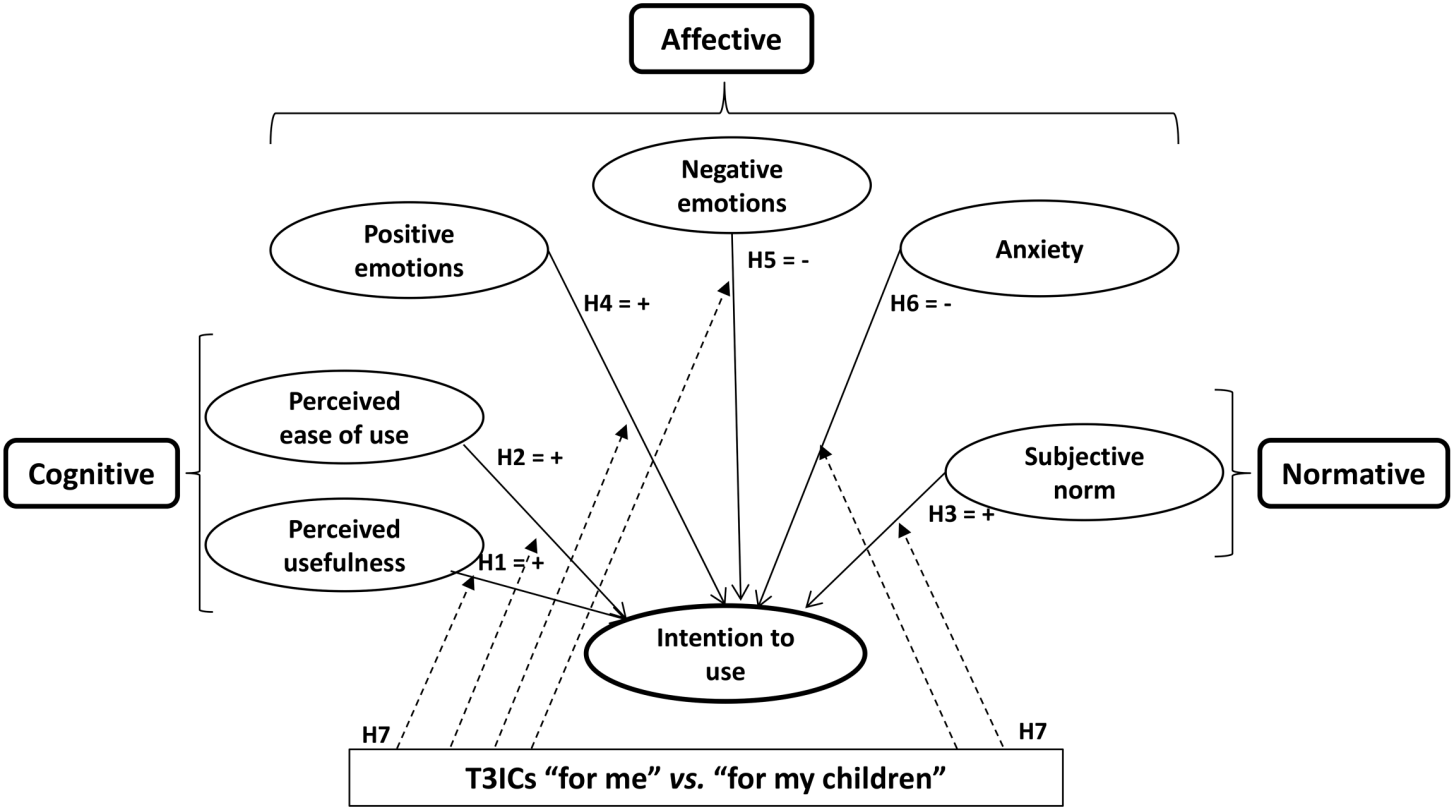
**Theoretical CAN model of acceptance of T3ICs “for me” vs. “for my child”**.

The formulated hypotheses define a proposal for a comprehensive theoretical model of variables influencing the intention to use T3ICs, namely, the CAN model shown in Figure 1.

## Methodology

### Data collection and sample characteristics

Data were retrieved from a self-administered, online survey. More than 3500 invitations to participate in the study were sent out. Only individuals over the age of 16 and residing in Spain could participate. The actual sample selected for the study consisted of 600 randomly selected individuals, proportionally distributed according to gender and age quotas. It is worth noting that the classification variable “implanted” and “non-implanted” participants was collected in the survey by the double question: *Do you have any implants?(due to cosmetic/health-related reasons) Of which type?* The percentage of implanted participants in the selected sample was however very low, some 8.7%. Full details are given in Table 1.

**Table 1:** **Technical details of the data collection and sample description**.

**DATA COLLECTION**		
Universe		Individuals over the age of 16
Sampling procedure		Stratified by gender and age
Data gathering		Self-administered, online survey (structured questionnaire)
Scope		Spain
Sample size		600 individuals
Fieldwork		April 2014
**SAMPLE CHARACTERISTICS**		
Gender	50% male, 50% female	
Age	≤20 years, 20%; 21–30 years, 20%; 31–40 years, 20%; 41–50 years, 20%; >51 years, 20%	

This study was approved by the Ethics Responsible at the Faculty of Business Administration of the University of La Rioja, and according to ICC/ESOMAR International Chamber of Commerce/ESOMAR (2008). Each participant provided informed consent.

### Statistical analysis

**Figure 2:**
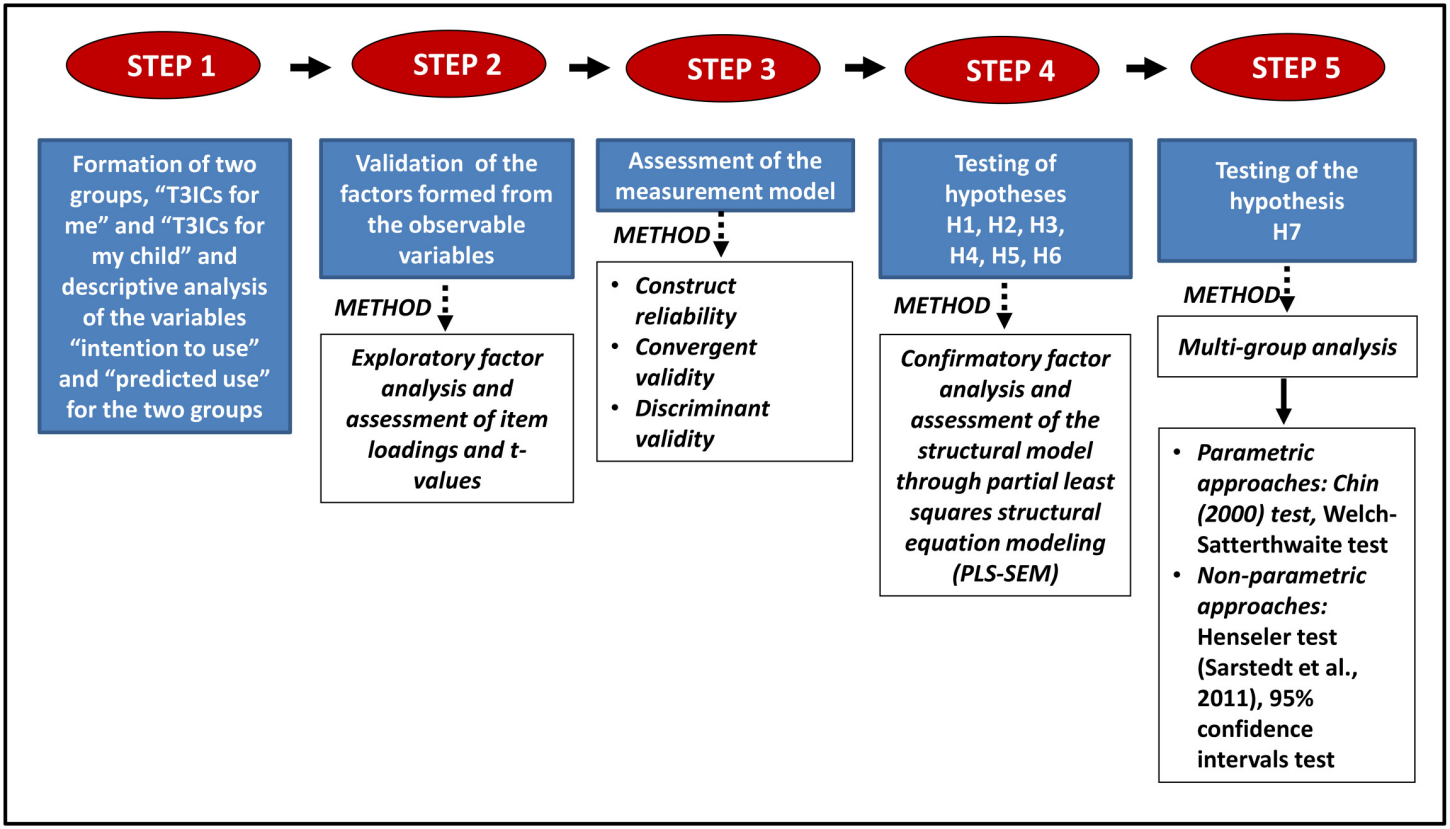
**Sequential statistical process**.

In order to test the working hypotheses, a sequential process was followed consisting of the following steps, which are summarized in Figure 2. Partial least squares structural equation modeling (PLS-SEM) was chosen to test the CAN model, as it is less sensitive to violations of data normality (Chin, 1998). The software used was SmartPLS 3.0.

- *Step 1: Formation of the two groups—“T3ICs for me” (Group 1) and “T3ICs for my child” (Group 2)—and descriptive analysis (mean, standard deviation, median, and paired sample tests of significant differences) of the variables “intention to use” and “predicted use” for the two groups*. The responses of each individual were separated into two groups (i.e., two datasets), one containing the responses referring to T3ICs “for me” and the other containing the same individual's responses regarding T3ICs “for my child.” Paired samples were obtained and a descriptive analysis of the variables “intention to use” and “predicted use” was performed.
- *Step 2: Exploratory factor analysis and validation of the factors formed from the observable variables*. To establish the factors formed from the observable variables a database was used that brought together the “T3ICs for me” and “T3ICs for my child” samples. In order to proceed to a multi-group comparison, the structure of the two models had to be identical. A joint model was built with the exact same factors and observable variables. The decision to eliminate certain observable variables from a factor was made based on this joint model.
- *Step 3: Assessment of the measurement model using PLS-SEM*. The measurement model was assessed by testing the reliability and validity of the measurement scales separately for each group.
- *Step 4: Assessment of the structural model, namely, testing of the hypotheses, using PLS-SEM. R*^2^, path coefficients, and their significance were estimated at this step. Hypotheses H1 to H6 were tested.
- *Step 5: Test for the multi-group comparison of the PLS models “T3ICs for me” (Model 1) and “T3ICs for my child” (Model 2)*. Both parametric and non-parametric tests were carried out to test the hypothesis of the existence of significant differences in the intention to use T3ICs in oneself and in one's child (H7). This hypothesis includes differences in the key relationships between Model 1 and Model 2, i.e., in the explanatory variables affecting the intention to use T3ICs, as well as in the intensity of their effect. Specifically, the parametric tests of Chin (2000) and Welch–Satterthwaite for similar and different variances between the two samples, respectively, were applied. The non-parametric tests applied were the Henseler test (Sarstedt et al., 2011) and the confidence intervals test.

## Results

### “Intention to use” and “predicted use” of “T3ICs for me” and “T3ICs for my child”

Table 2 shows the descriptive mean, standard deviation, and median for the variables “intention to use” and “predicted use” and the groups T3ICs “for me” and T3ICs “for my child.” There are significant differences in the mean values for the “for me” and “for my child” groups. The mean values for “intention to use” and “predicted use” for the “T3ICs for me” group were around 4.6 (on a scale of 0–10). These mean values were lower for the “T3ICs for my child” group (around 3.9). However, there was a high dispersion of mean values for “intention to use” and “predicted use” for both groups (close to 3.3 in both cases). Median values were therefore estimated, which differ by one point.

**Table 2:** **“Intention to use” and “predicted use” of T3ICs “for me” and T3ICs “for my child”**.

	**Intention to use**			**Predicted use**		
	**Mean**	**SD**	**Median**	**Mean**	**SD**	**Median**
T3ICs “for me”	4.64	3.38	5.00	4.61	3.38	5.00
T3ICs “for my child”	3.96	3.33	4.00	3.86	3.28	4.00
*p*-value paired sample *t*-test	*p* < 0.001			*p* < 0.001		
*p*-value Wilcoxon test (paired samples)	*p* < 0.001			*p* < 0.001		

*Mean, standard deviation (SD), and median. SD stands for standard deviation*.

Together, the results show that the acceptance of T3ICs is higher when they are for oneself than when they are for one's child. Moreover, the high dispersion of mean values for both groups justifies testing the proposed model for explaining the acceptance of T3ICs and the differences found for this acceptance between the “T3ICs for me” and “T3ICs for my child” groups.

### Exploratory factor analysis and validation of the factors formed from the observable variables

Exploratory factor analysis was carried out to test the factors naturally formed from the observable variables, i.e., the measurement scales. Based on the results from the exploratory factor analysis, “PU”, “PEU”, “subjective norm” (SN), and “intention to use” (IU) were all formed of a single factor with high variance explained: PU = 91.90% (KMO = 0.845), PEU = 91.97% (KMO = 0.875), SN = 97.71% (KMO = 0.500), and IU = 96.46% (KMO = 0.500). Bartlett's sphericity tests were significant for all the aforementioned scales (*p* < 0.001).

The affective scale, however, was more complex. This scale was formed of three differentiated factors, “positive emotions,” “negative emotions,” and “anxiety,” which together explained 73.15% of the variance in the intention to use T3ICs. The KMO index was 0.941, and the Bartlett's sphericity test was significant (*p* < 0.001). “Positive emotions” included feeling enthusiastic, determined, proud, inspired, strong, active, interested, and excited, that is, positive feelings toward the use of T3ICs. “Negative emotions” included feeling hostile, upset, irritable, ashamed, and guilty. Finally, “anxiety” involved being afraid, scared, jittery, alert, nervous, distressed, and attentive.

### Assessment of the measurement model

The assessment of the measurement model was carried out in two steps. First, item validity was examined. This was assessed in terms of the standardized loadings (>0.70) and *t*-values (>1.96) (Hair et al., 2013). The latter indicates the significant contribution of a variable to the content validity of the corresponding factor. Exceptionally, a significant variable can be kept in the model to the detriment of the standardized loading (Hair et al., 2013). Based on these criteria, it was decided to remove the variables “alert” and “attentive” (not significant) and to preserve the variable “ashamed” (standardized loading of 0.690 and *t*-value of 12.448 for the “T3ICs for me” group, and of 0.744 and 13.384, respectively, for the “T3ICs for my child” group). The standardized loadings and *t*-values of all the variables included in the final model are shown in the Appendix.

Second, the measurement model was verified in terms of construct reliability (i.e., composite reliability and Cronbach's Alpha), convergent validity, and discriminant validity. The composite reliability and Cronbach's Alpha values were all above 0.70. The convergent validity of the constructs was also satisfied, with an average variance explained (AVE) above 0.5 in all cases. The discriminant validity of the constructs was measured through the comparison of the square root of AVE vs. the correlations among constructs (Roldán and Sánchez-Franco, 2012). The square root of AVE (diagonal elements in bold in Table 3) has to be larger than the corresponding inter-construct correlations (off-diagonal elements in Table 3). This criterion was also met in all cases.

**Table 3:** **Construct reliability, convergent validity, and discriminant validity of T3ICs “for me” and T3ICs “for my child”**.

**Construct**	**CR (>0.70)**	**Cronbachs' Alpha (>0.70)**	**AVE (>0.50)**	**PU**	**PEU**	**SN**	**PE**	**NE**	**A**	**IU**
**T3ICs “FOR ME”**										
Perceived usefulness (PU)	0.98	0.97	0.92	**0.96**						
Perceived ease of use (PEU)	0.98	0.97	0.92	0.66	**0.96**					
Subjective norm (SN)	0.97	0.95	0.95	0.45	0.34	**0.97**				
Positive emotions (PE)	0.95	0.94	0.72	0.64	0.54	0.56	**0.85**			
Negative emotions (NE)	0.90	0.86	0.65	−0.17	−0.13	−0.05	−0.06	**0.81**		
Anxiety (A)	0.92	0.91	0.69	−0.09	−0.06	−0.06	0.04	0.74	**0.83**	
Intention to use (IU)	0.99	0.97	0.97	0.63	0.54	0.69	0.75	−0.19	−0.27	**0.99**
**T3ICs “FOR MY CHILD”**										
Perceived usefulness (PU)	0.98	0.97	0.92	**0.96**						
Perceived ease of use (PEU)	0.98	0.97	0.92	0.66	**0.96**					
Subjective norm (SN)	0.99	0.98	0.98	0.41	0.32	**0.99**				
Positive emotions (PE)	0.95	0.94	0.72	0.48	0.40	0.60	**0.85**			
Negative emotions (NE)	0.92	0.88	0.71	−0.11	−0.07	−0.05	0.05	**0.84**		
Anxiety (A)	0.93	0.93	0.73	−0.06	−0.03	−0.07	0.07	0.77	**0.85**	
Intention to use (IU)	0.99	0.98	0.98	0.48	0.40	0.80	0.72	−0.13	−0.19	**0.99**

*CR stands for composite reliability. AVE stands for average variance explained. Diagonal elements (in bold) are the square root of the AVE. Off-diagonal elements are the inter-construct correlations*.

### Assessment of the structural model

The CAN model greatly explained the intention to use T3ICs. The *R*^2^ was 73.8% for T3ICs “for me” and 75.9% for T3ICs “for my child” (Table 4). Stone-Geisser's cross-validated redundancy Q^2^ was >0 in both cases, specifically, 0.718 for T3ICs “for me” and 0.740 for T3ICs “for my child.” These results further confirmed the predictive relevance of the CAN model (see Hair et al., 2011a,b). The variance explained by each factor and for each group are also shown in Table 4.

**Table 4:** **Effect on endogenous variables**.

	***R*^2^**	***Q*^2^**	**Direct effect**	**Correlation**	**Variance explained (%)**
**T3ICs “FOR ME”**					
Intention to use	73.8%	0.718			
H1: Perceived usefulness = > (+) Intention to use			0.082	0.626	5.13
H2: Perceived ease of use = > (+) Intention to use			0.114	0.544	6.20
H3: Subjective Norm = > (+) Intention to use			0.365	0.692	25.19
H4: Positive emotions = > (+) Intention to use			0.426	0.752	31.88
H5: Negative emotions = > (−) Intention to use			−0.162	−0.274	4.44
H6: Anxiety = > (+) Intention to use			−0.051	−0.187	0.95
**T3ICs “FOR MY CHILD”**					
Intention to use	75.9%	0.740			
H1: Perceived usefulness = > (+) Intention to use			0.037	0.480	1.78
H2: Perceived ease of use = > (+) Intention to use			0.041	0.397	1.63
H3: Subjective Norm = > (+) Intention to use			0.532	0.798	42.45
H4: Positive emotions = > (+) Intention to use			0.373	0.718	26.78
H5: Negative emotions = > (−) Intention to use			−0.168	−0.194	3.26
H6: Anxiety = > (+) Intention to use			0.003	−0.128	−0.04

**Figure 3:**
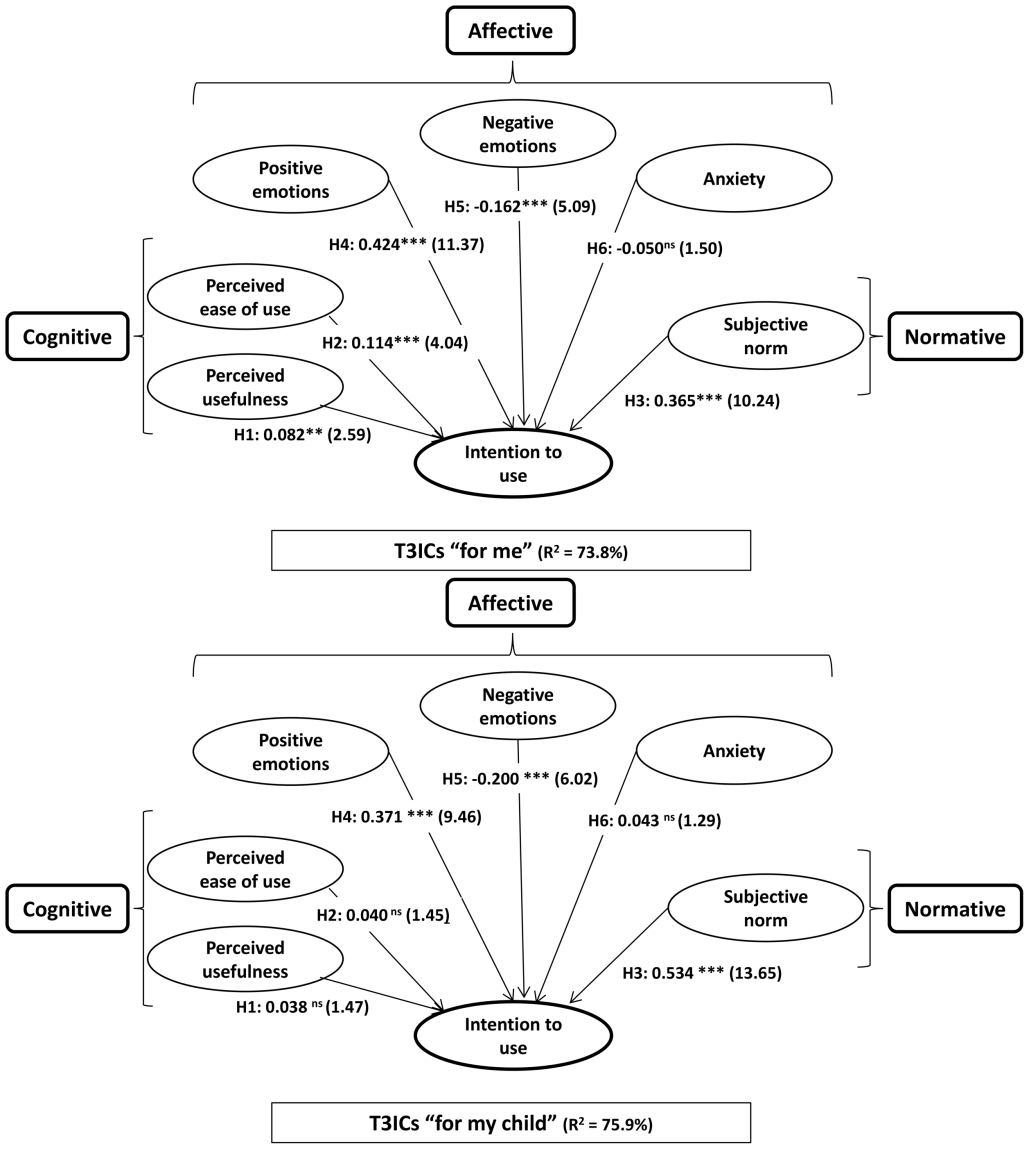
**Sign, magnitude, and significance of the path coefficients, and ***R***^**2**^ of T3ICs “for me” and T3ICs “for my child.”** ^*^p < 0.05 => *t* > 1.65; ^**^p < 0.01 => *t* > 2.33; ^***^p < 0.001 => *t* > 3.09; n.s. = not significant [based on *t*_(4.999)_, one-tailed test]. Please note that the levels of significance (*p*-values) are either “non significant” or lower than 0.01.

The sign, magnitude, and significance of the path coefficients and the *R*^2^ are shown in Figure 3 and Table 5. Three hypotheses were fully supported by the results: H3 (regarding the influence of the “subjective norm”), H4 (regarding “positive emotions”), and H6 (regarding “negative emotions”) (Table 5). These relationships were significant in both models, and the direction set coincided with that hypothesized. Two hypotheses were partly supported: H1 and H2. Cognitive factors affected the intention to use the technology on oneself but not on one's child. The relationship was only significant, and only coincided with the direction set, in Model 1 (“T3ICs for me”). H5 (regarding “anxiety”) was rejected, as the relationship was not significant in either group.

**Table 5:** **Path coefficients, ***t***-values, and support for the hypotheses**.

**Hypothesis**	**T3ICs “for me”**		**T3ICs “for my child”**		**Support for hypothesis**
	**Path coefficients**	***t*-value**	**Path coefficients**	***t*-value**	
H1: Perceived usefulness = > (+) Intention to use	0.082^**^	2.59	0.038^n.s.^	1.47	Partly supported
H2: Perceived ease of use = > (+) Intention to use	0.114^***^	4.04	0.040^n.s.^	1.45	Partly supported
H3: Subjective norm = > (+) Intention to use	0.365^***^	10.24	0.534^***^	13.65	Supported
H4: Positive emotions = > (+) Intention to use	0.424^***^	11.37	0.371^***^	9.46	Supported
H5: Negative emotions = > (−) Intention to use	−0.162^***^	5.09	−0.20^***^	6.02	Supported
H6: Anxiety = > (−) Intention to use	−0.050^n.s.^	1.50	0.043^n.s.^	1.29	Rejected

^*^p < 0.05 = > t > 1.65;

** *p < 0.01 = > t > 2.33*;

*** *p < 0.001 = > t > 3.09*;

*n.s. = not significant [based on t_(4.999)_, one-tailed test]. Please note that the levels of significance (p-values) are either “non significant” or lower than 0.01*.

### Multi-group analysis

A multi-group analysis was performed to compare the results of the models for each group. Two parametric and two non-parametric tests were used to analyze differences in the key relationships between the models and to further assess possible moderating effects (Tables 6, 7).

**Table 6:** **Multi-group comparison**.

**Hypothesis**	**Difference “for me” – “for my child”**	**P_EV_**	**P_W-S_**	**P_*H*_**
H1: Perceived usefulness = > (+) Intention to use	0.044	0.270	0.270	0.135
H2: Perceived ease of use = > (+) Intention to use	0.073	0.060	0.060	0.029
H3: Subjective norm = > (+) Intention to use	−0.169	0.002	0.002	0.001
H4: Positive emotions = > (+) Intention to use	0.052	0.336	0.336	0.169
H5: Negative emotions = > (−) Intention to use	0.007	0.801	0.801	0.401
H6: Anxiety = > (−) Intention to use	−0.054	0.209	0.209	0.104

*Levels of significance based on Student t_(4.999)_ distribution with two tails. P_EV_ = p-value equivalent variances test. P_W−S_ = p-value Welch–Satterthwaite test. P_H_ = p-value Henseler test*.

**Table 7:** **Non-parametric test of confidence intervals and multi-group comparison**.

**Hypothesis**	**Confidence intervals**		**Significance**
	**“For me” (2.5%, 97.5%)**	**“For my child” (2.5%, 97.5%)**	
H1: Perceived usefulness = > (+) Intention to use	(0.085, 0.142)	(−0.015, 0.016)	n.s.
H2: Perceived ease of use = > (+) Intention to use	(0.096, 0.172)	(−0.009, 0.061)	n.s.
H3: Subjective norm = > (+) Intention to use	(0.607, 0.434)	(0.454, 0.289)	sig.
H4: Positive emotions = > (+) Intention to use	(0.446, 0.497)	(0.294, 0.351)	n.s.
H5: Negative emotions = > (−) Intention to use	(−0.089, −0.100)	(−0.225, −0.222)	n.s.
H6: Anxiety = > (−) Intention to use	(0.058, 0.008)	(−0.080, −0.119)	n.s.

*Sig. denotes a significant difference at 0.05; n.s. denotes a non-significant difference at 0.05*.

Column P_EV_ in Table 6 shows the *p*-values obtained applying the method proposed by Chin (2000). This method assumes that the data is normally distributed and/or that the variances of the two samples are similar (Afonso et al., 2012). Column P_W−S_ shows the *p*-values obtained applying the Welch–Satterthwaite test in the cases where the variances of the two samples were different. The results of these two parametric tests were similar.

As for the non-parametric tests (Sarstedt et al., 2011), column P_H_ in Table 6 shows the *p*-value obtained applying the Henseler test. Table 7 shows the results of the test of confidence intervals, the second non-parametric test used. The criteria establish that when the parameters estimated through confidence intervals for the two groups overlap, a significant difference can be established between the two group-specific path coefficients.

In three out of the four tests performed, a significant difference was found between the two groups regarding the key relationship between “PEU” and “intention to use”; the exception was the result of the confidence intervals test. However, because the confidence intervals test is relatively more conservative than the other three tests (Sarstedt et al., 2011), the significance between the groups of the difference in the relationship between “PEU” and “intention to use” was accepted. The relationship between “subjective norm” and “intention to use” was significantly different according to the four tests performed. No significant differences were found in other key relationships.

## Discussion, implications, limitations, and future research

This study introduces a model that combines cognitive, affective, and normative factors to explain the acceptance of a new insideable technology, namely, technological implants to increase innate human capacities (T3ICs). The CAN model largely explains the intention to use the technology for the specified groups, with a variance explained (*R*^2^) of 73.8% for Group 1 (“T3ICs for me”) and of 75.9% for Group 2 (“T3ICs for my child”). The variables contributing the most were found to be “positive emotions” and (positive) “subjective norm.”

The CAN model is based on previous models of technology acceptance, specifically, TAM models (Davis, 1989; Davis et al., 1989; Venkatesh and Davis, 2000) and UTAUT models (Venkatesh et al., 2003, 2012). These models merely include cognitive and normative variables. Venkatesh et al. (2012) have contributed significantly to the application of these models, developing the UTAUT model, which obtained a PLS *R*^2^ of 44% for direct effects. This value compares to the substantially higher *R*^2^ obtained through the inclusion of the emotional dimension in the CAN model: *R*^2^ above 70% for both of the specified groups. These results thus confirm the benefits of extending the factors determining the acceptance of a new technology to include the emotional dimension of consumer behavior. Affective factors greatly contribute to explaining underlying motives influencing subjects' assessment of products (Pieters and van Raaij, 1988; van Waterschoot et al., 2008; Levav and McGraw, 2009; Zielke, 2011) through variables such as “positive emotions,” “negative emotions,” and “anxiety.”

The results showed that the acceptance of T3ICs was higher when the intended recipient was oneself than when it was one's child. Moreover, statistically significant differences were found between the two models/groups—“T3ICs for me” and “T3ICs for my child”—when applying the multi-group comparison to the three dimensions specified in the CAN model, namely, the cognitive, affective, and normative dimensions.

The cognitive variables “PU” and “ease of use” influenced the intention to use T3ICs on oneself, but not on one's child. That is, neither “PU” nor “ease of use” had a (positive) significant effect on the intention to have one's child implanted (H1 and H2 were partly supported). The between-groups comparison yielded statistically significant differences regarding the variable “PEU” in at least three of the four multi-group tests applied. The moderating effect of the end user in this relationship was thus accepted. It is worth noting, however, that significant differences between the groups were not found with regard to the variable “PU.” The reason for this is two-fold: first, the scant contribution of this variable to overall variance explained (only 5.13% in the reference group “T3ICs for me”), and second, the equally low difference in standardized loadings between the two groups (only 0.044 points).

In conclusion, the cognitive variables included in the CAN model have only a limited influence when the end user is oneself, and no influence when the end user is one's child. The results slightly modify those of previous studies, such as Li et al. (2003), Christiansen and Leigh (2004), and Christie and Bloustien (2010), that have shown the importance of “PU” in the decision to implant one's child for health-related reasons. In the current study, implantation is not performed for medical reasons, and this variable ceases to have an effect. Likewise, it can be concluded that “PU” is a relevant factor in the decision of whether to get a T3IC, but is not as essential a factor as it is in the decision of whether to undergo cosmetic surgery (see Adams, 2010).

Regarding the normative dimension, in both groups the variable “subjective (or social) norm” positively influenced the intention to use T3ICs (H3 was supported). Moreover, the four multi-group analyses revealed significant differences between the two groups for this normative variable. This variable largely explains the intention to use T3ICs in the “for my child” group (42.45%, the highest variance explained), while it is the second variable in terms of the percentage of variance explained (25.19%) for the “for me” group. These results are consistent with those of von Soest et al. (2006), Most et al. (2007), Hyde et al. (2010), Adams (2010), Javo and Sørlie (2010), and Dorneles de Andrade (2010), who established that family, friends, and society influence the decision to undergo changes in the body. However, the current study has shown that this influence is stronger when the decision affects one's child than when it affects oneself.

As for the emotional dimension, both positive and negative emotions affected the intention to use T3ICs in the direction established in the CAN model. H4 and H5 were thus supported. In both groups, positive emotions explained the intention to use T3ICs (31.88% for the “T3ICs for me” group and 26.78% for the “T3ICs for my child” group). Negative emotions explained the intention to use them in both groups to a lesser extent (4.44% and 3.26%, respectively). The multi-group analysis showed no significant differences between the two groups regarding the influence of these two variables on the intention to use T3ICs. These findings support studies establishing that positive emotions promote a positive assessment of a technology (Bagozzi, 1997; Shiv and Fedorikhin, 1999). However, the proposal of a natural tendency to make decisions that minimize the probability of negative emotions occurring (Elliott, 1998; Schwarz, 2000; Han et al., 2007) was found to have little bearing, at least in this case.

The influence of “anxiety” on the intention to use T3ICs was non-significant in the models of both groups (H6 was rejected). The results of the multi-group analysis showed no differences between the two groups. This lack of influence of “anxiety” is contrary to the research of Buchanan-Oliver and Cruz (2011), which determined the anxiety produced by the idea of the dissolution of the limits of what is human due to the introduction of implants. However, it is consistent with what Venkatesh et al. (2003) demonstrated when they developed the UTAUT model.

Finally, as detailed before, differences were found between the two groups for some of the key relationships specified in the CAN model. H7 can thus be partly accepted. Another contribution derived from the results of this study is related to the demonstration of the moderating effect of the end user on the acceptance of a new technology. When one is considering the decision to get T3ICs for one's child, social influence is the principal factor. At a considerable distance from the “social norm,” emotions, especially “positive emotions,” have a secondary level of importance. In this case, it did not matter whether the T3ICs were considered to be useful or easy-to-use; neither of these variables significantly explained the intention to use T3ICs. When the T3ICs were for oneself, “positive emotions” were what most greatly explained the intention to use T3ICs, followed closely by “social norm.”

The CAN model proved useful for explaining the intention to use a technology in the early stages of adoption; however, the importance of the variables in explaining such an intention to use varied depending on the moderating variable “for me” vs. “for my child.” When studying the acceptance of a technology, one should thus distinguish between when the recipient is oneself and when it is another user. Lastly, the low explanatory power of cognitive variables may be due precisely to the early stage of development in which the studied technology currently finds itself. Consumers know very little about the usability of the products and are more concerned about what they feel and what others will think of them (i.e., social norm).

### Implications of the results

This work opens a new line of research on the acceptance of a technology integrated into the human body with psycho-sociological implications for the evolution toward a human with superior capacities. Challenges for companies selling T3ICs involve two main aspects, convincing society of the goodness of T3ICs and generating positive emotions toward this type of product. When parents are deciding on getting T3ICs for their children, the battle must be won by convincing society of the goodness of these implants, since the social norm is the most important aspect. On the other hand, when the decision is about whether or not to implant oneself, the development of marketing communications generating positive emotions toward T3ICs would be the most effective way forward. Encouraging the idea that T3ICs are useful and easy-to-use, while important, may be of secondary concern. Nonetheless, sooner or later companies will have to address these barriers and opportunities.

### Limitations and future research

So far, the CAN model has been applied to the general idea of T3ICs. However, the results could vary if it were applied to a particular type of T3IC. “PU” and “ease of use” could, for instance, acquire more relevance. Therefore, as a future line of research, the CAN model could be applied to specific types of T3ICs to observe consumers' reactions. In addition, the model was tested on an emerging product, and it was not possible to ascertain whether its explanatory power would be similarly high for more widely used technology products. The CAN model should thus be tested on these and other products with the same degree of diffusion, as well-known types of implants would most likely be assessed differently, a factor that might also depend on respondents' technological literacy (see Schaar and Ziefle, 2011). In this regard, cultural factors might be also relevant, since different countries have different degrees of technological proneness and literacy (Alagöz et al., 2011). Other factors worth considering are gender and age differences in attitudes and acceptance of technological implants, also in relation to general attitudes toward technology (self-reported technological interest, literacy, handling competence, and distrust in technology) (Ziefle and Schaar, 2011). Moreover, future research could focus on analyzing the differences on the acceptability of T3ICs depending on whether individuals have already undergone implantation due to cosmetic/health-related reasons. This would, however, involve a new sample including a larger percentage of implanted participants.

With regard to the decision to implant a third party, the current study was limited to comparing the acceptance of implants “for me” vs. “for my child.” However, several companies in the US already use insideable tech to identify their workers. Moreover, one proposed public health system in the US also contemplates the possibility of requiring people to get ID implants. Such events have generated public controversy. In this regard, another possible line of research would be to examine the reactions arising from having the decision to undergo implantation be imposed by an external authority.

Finally, the current study does not take the ethical component into account. The authors believe that these types of products could greatly exacerbate social differences. A society could emerge made up of an implanted elite alongside non-implanted children who would be unable to compete to reach the same levels of development as their implanted counterparts. It is thus essential for future research dealing with this issue to return as much information as possible to society in order to enable the type of informed decision-making that will be essential to our progress as social human beings.

## Author contributions

The directors of research have been the professors JP, ER, and CO. The three co-authors have participated in all stages of work, including the conception and design of the research, the revision of intellectual content and drafting the work. MG has participated in the revision of intellectual content and the drafting and translation of the article.

### Conflict of interest statement

The authors declare that the research was conducted in the absence of any commercial or financial relationships that could be construed as a potential conflict of interest.
