# Supplementary material for: Assessing the Moderating Effect of the End User in Consumer Behavior: The Acceptance of Technological Implants to Increase Innate Human Capacities
Source: Front Psychol. 2016 Feb 22;7:132. doi: 10.3389/fpsyg.2016.00132 (PMC4761839; doi:10.3389/fpsyg.2016.00132)
Supplement: Supplementary file 1 [file DataSheet1.pdf]

Appendix

Table A1 shows the results of the assessment of the measurement model, namely, the standardized loadings and t-values of all the variables included in the final CAN model.

TABLE A1 Standardized loadings and t-values of T3ICs "for me" and T3ICs "for my child."

	T3ICs "for me"		T3ICs "for my child"	
	Loading	t-value	Loading	t-value
Intention to use				
Intention to use	0.987	488.144	0.989	489.793
Predicted use	0.987	454.374	0.990	517.188
Perceived usefulness				
Performance	0.961	222.588	0.961	233.979
Productivity	0.964	180.862	0.964	177.831
Effectiveness	0.961	172.136	0.961	173.109
Usefulness	0.949	145.696	0.949	132.411
Perceived ease of use				
Easy to learn	0.953	143.988	0.952	136.070
Easy to use	0.940	100.977	0.942	112.618
Easy to become skillful	0.971	177.777	0.971	176.604
Easy to do	0.971	240.338	0.971	231.515
Subjective norm				
Influence me	0.975	204.883	0.988	472.822
Important to me	0.976	248.345	0.988	467.299
Positive emotions				
Interested	0.813	49.268	0.803	47.387
Proud	0.859	67.068	0.865	54.907
Inspired	0.861	58.415	0.828	43.154
Strong	0.864	57.748	0.875	59.352
Active	0.850	54.432	0.862	54.538
Excited	0.739	25.190	0.774	29.287
Determined	0.871	65.755	0.866	59.264
Enthusiastic	0.932	157.869	0.911	79.512
Negative emotions				
Irritable	0.868	39.641	0.888	38.565
Ashamed	0.690	12.448	0.744	13.384
Upset	0.888	53.377	0.896	51.561
Guilty	0.688	12.431	0.791	17.456
Hostile	0.873	52.063	0.881	40.377
Anxiety				
Nervous	0.728	6.980	0.771	3.719
Jittery	0.780	8.338	0.745	3.556
Distressed	0.848	18.615	0.917	4.907
Afraid	0.899	17.799	0.894	5.225
Scared	0.902	18.784	0.928	5.424
